# Determination of Cd^2+^ and Pb^2+^ Based on Mesoporous Carbon Nitride/Self-Doped Polyaniline Nanofibers and Square Wave Anodic Stripping Voltammetry

**DOI:** 10.3390/nano6010007

**Published:** 2016-01-04

**Authors:** Chang Zhang, Yaoyu Zhou, Lin Tang, Guangming Zeng, Jiachao Zhang, Bo Peng, Xia Xie, Cui Lai, Beiqing Long, Jingjing Zhu

**Affiliations:** 1 College of Environmental Science and Engineering, Hunan University, Changsha 410082, China; zhangchang@hnu.edu.cn (C.Z.); iluenjor@163.com (Y.Z.); pengbo_hnu@163.com (B.P.); xxialmf@163.com (X.X.); laicui@hnu.edu.cn (C.L.); longbeiqing@126.com (B.L.); metzelsuppe@163.com (J.Z.); 2 Key Laboratory of Environmental Biology and Pollution Control, Ministry of Education, Hunan University, Changsha 410082, China; 3 College of Resources and Environment, Hunan Agricultural University, Changsha 410128, China; jiachao.zhang@163.com

**Keywords:** sensor, heavy metals, mesoporous carbon nitride, polyaniline, electrochemistry

## Abstract

The fabrication and evaluation of a glassy carbon electrode (GCE) modified with self-doped polyaniline nanofibers (SPAN)/mesoporous carbon nitride (MCN) and bismuth for simultaneous determination of trace Cd^2+^ and Pb^2+^ by square wave anodic stripping voltammetry (SWASV) are presented here. The morphology properties of SPAN and MCN were characterized by transmission electron microscopy (TEM), and the electrochemical properties of the fabricated electrode were characterized by cyclic voltammetry (CV). Experimental parameters, such as deposition time, pulse potential, step potential, bismuth concentration and NaCl concentration, were optimized. Under the optimum conditions, the fabricated electrode exhibited linear calibration curves ranging from 5 to 80 nM for Cd^2+^ and Pb^2+^. The limits of detection (LOD) were 0.7 nM for Cd^2+^ and 0.2 nM for Pb^2+^ (S/N = 3). Additionally, the repeatability, reproducibility, anti-interference ability and application were also investigated, and the proposed electrode exhibited excellent performance. The proposed method could be extended for other heavy metal determination.

## 1. Introduction

Cadmium(II) and lead(II) are severe hazardous environmental pollutants with toxic effects on living organisms due to their non-biodegradability and persistence [1,2]. For example, lead has obvious effects on renal, brain development and blood pressure [3], and cadmium is primarily toxic to the kidney, especially to the proximal tubular cells [4]. Therefore, it is of significance to develop sensitive, rapid and simple analytical methods for the detection of trace amounts of Pb^2+^ and Cd^2+^ ions [5,6]. The common methods for heavy metals analysis include atomic absorption spectrometry (AAS) [7], atomic emission spectrometry (AES) [8], atomic fluorescence spectrometry [9] and inductively-coupled plasma mass spectrometry (ICP-MS) [10]. However, the widespread use of these techniques is limited due to the relatively costly and difficult for the field on-line analysis.

In contrast, electrochemical techniques for heavy metals detection offer several advantages, such as low-cost, simplicity, accurateness, remarkable sensitivity, high stability, suitability for multiplexed detection and the capability of on-line environmental monitoring [11,12,13]. Anodic stripping voltammetry (ASV) has been considered to be the most effective tool for the quantification of trace metal ions due to an effective pre-concentration step followed by electrochemical stripping measurements of the accumulated analytes. Recently, the bismuth-based chemically-modified electrodes (CMEs) have been proven to be highly sensitive and reliable for trace analysis of Cd^2+^ and Pb^2+^ in conjunction with anodic stripping voltammetry (ASV), due to the unique behavior of bismuth nano-modified electrodes being attributed to the formation of multi-components alloys, as well as the enhanced sensibility coming from the combination of the great properties of the nanostructured material [11,14,15].

Therefore, unique nanostructures of advanced materials, such as quantum dots [16], metal nanoparticles [17], grapheme [18], carbon nanotubes [19], and so on, have been developed for bismuth-based electrochemical sensors. Ordered mesoporous carbon nitride material (MCN) with large surface areas, small particle sizes, tunable pore diameters and high biocompatibility and activity because of the CN matrix promises access to and good performances in a wider range of applications, such as catalyst, supercapacitors, adsorbent, and so on. In our previous study, ordered mesoporous carbon nitride (MCN) was used as the platform for electrochemical sensors, which can obviously increase the sensitivity and lower the detection limit for phenols and heavy metals [20,21]. MCN has faster electron transfer between substrate and MCN-sensing sites because of the π-π* electronic transition in the MCN. Therefore, MCN was exploited as a platform to convert the recognition information into a detectable signal. In addition, polyanilines (PAN) have unique properties, such as, high electrical conductivity, good environmental stability, facile synthesis, low cost, homogeneity, special redox properties and strong adherence to the electrode surface, and thus, PAN has been extensively applied in electrochemical sensors [22]. However, the conductivity of PAN will be significantly reduced when the pH of the solution is higher than four [23], which largely limits its application in sensors. Fortunately, to overcome this drawback, many kinds of PAN derivatives, such as self-doped polyanilines (SPANs), have been synthesized by different methods and used as a new electroactive material for H_2_O_2_ and DNA detection [24,25,26] and a high performance redox supercapacitor [27]. Compared to the parent PAN, SPAN with the functional groups, such as sulfonic and carboxylic acid, owns their unique properties. Especially, SPAN shows an extended pH range for electric conductivity and electrochemical activity, as well [28], which improved the potential to determine Cd^2+^ and Pb^2+^ levels in real samples.

In this paper, combining the advantages of MCN and SPAN nanofibers, we developed a highly sensitive sensor for electrochemical analysis of trace Cd^2+^ and Pb^2+^ through square wave anodic stripping voltammetry (SWASV), which has rarely been reported. The SPAN nanofibers and MCN-modified glassy carbon electrode (GCE) showed a remarkable capability of faster electron transfer and excellent stability. At the same time, bismuth nanoparticles were used for trace analysis of heavy metals in conjunction with anodic stripping voltammetry. In a word, this sensor exhibited outstanding performance in cost, simplicity, accurateness, sensitivity and stability and has certain practical significance.

## 2. Results and Discussion

### 2.1. Morphology Characterization of SPAN Nanofibers and MCN

**Figure 1:**
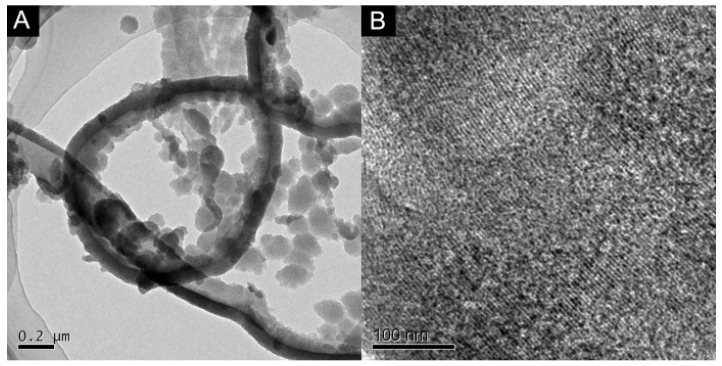
Transmission electron microscopy (TEM) of the (**A**) self-doped polyaniline (SPAN) nanofiber and (**B**) mesoporous carbon nitride (MCN).

The morphology characterization of SPAN nanofibers was investigated by TEM. As shown below, Figure 1A shows that SPAN nanofibers were interconnected through many particles to form a net-like or ring-like structure, which contributed to the uniform modification of the electrode surface by MCN. Besides, from the TEM image of Figure 1B, a highly order stripe-like structure can be observed clearly.

### 2.2. Electrochemical Behavior

**Figure 2:**
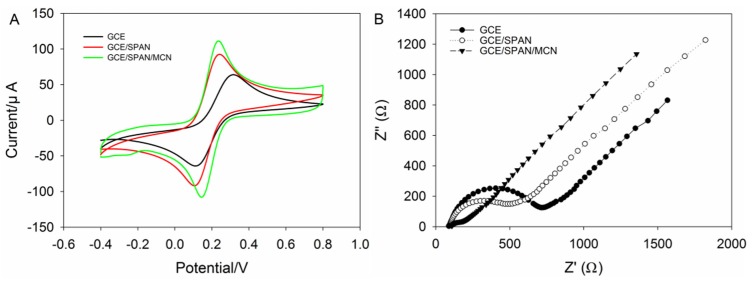
(**A**) Cyclic voltammetry diagrams of the glassy carbon electrode (GCE), GCE/SPAN and GCE/SPAN/MCN, using a 0.1 M KCl solution containing 5.0 mM ferro-/ferri-cyanide, with a potential range of −0.4–0.8 V and a scan rate of 100 mV·s^−1^; (**B**) electrochemical impedance spectra of GCE, GCE/SPAN and GCE/SPAN/MCN using a 0.1 M KCl solution containing 5.0 mM ferro-/ferri-cyanide, with a frequency range of 0.1–10^5^ Hz, a bias potential of 0.19 V *vs.* a saturated calomel electrode (SCE) and an alternating current (AC) amplitude of 5 mV.

Cyclic voltammetry (CV) and electrochemical impedance spectroscopy (EIS) were used to investigate the characteristics of different modified electrodes (bare GCE, GCE/SPAN and GCE/SPAN/MCN). CV was carried out in 5.0 mM [Fe(CN)_6_]^3−/4−^ (1:1) solution containing 0.1 M KCl for different modified electrodes, shown in Figure 2A. As can be seen, compared to the bare GCE, both GCE/SPAN and GCE/SPAN/MCN showed relatively larger current signals. The reason for that might be analyzed from the fact that the synergistic amplification effect of the SPAN nanofibers and MCN accelerates the electron transfer of [Fe(CN)_6_]^3−/4−^ on the MCN/SPAN membrane. Besides, the CVs indicated that the modified electrode has a good current response performance.

EIS of [Fe(CN)_6_]^3−/4−^ is used to provide information about the interface properties and impedance changes in the process of electrode modification [29]. It is well known that in the typical Nyquist plot of impedance spectra, a semicircle portion at higher frequencies corresponds to the electron transfer resistance, and a linear portion at lower frequencies corresponds to the diffusion process [30]. Figure 2B exhibits the Nyquist diagrams of the different modified electrodes, and the inset depicts the equivalent circuit. According to the method described in our previous report [21], the bare GCE has a relative large electron-transfer resistance (*R*_ct_) value of about 778.4 Ω. The *R*_ct_ value had a very small increase after modification of SPAN. An almost straight line was obtained after modification by MCN with superior electrical conductivity, indicating that MCN can accelerate the electron transfer on the electrode. This increase indicated that the SPAN and MCN were successfully modified with GCE, and they provided a large surface area and more binding sites with heavy metal ions, as well as accelerate the electron transfer.

### 2.3. Optimization of Experimental Parameters

#### 2.3.1. Optimization of Supporting Electrolyte

**Figure 3:**
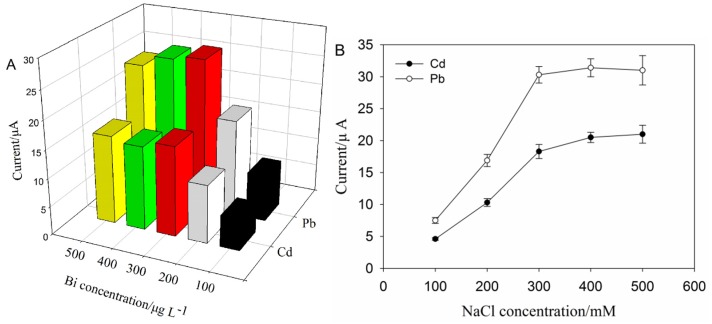
(**A**) Bi^3+^ concentration effect on peak height in a solution containing 200 mM NaCl, 10 μg·L^−1^ Pb^2+^ and Cd^2+^. Square wave anodic stripping voltammetry (SWASV) parameters: *E*_begin_ = −1 V, *E*_end_ = −0.4 V, *E*_step_ = 0.010 V, *E*_pulse_ = 0.02 V, *E*_condition_ = −0.4 V, *E*_deposition_ = −1 V, frequency = 50 Hz, deposition time = 300 s and equilibrium time = 20 s. (**B**) NaCl concentration effect on peak height in a solution containing 300 μg·L^−1^ Bi^3+^, 10 μg·L^−1^ Pb^2+^ and Cd^2+^. SWASV parameters: *E*_begin_ = −1 V, *E*_end_ = −0.4 V, *E*_step_ = 0.010 V, *E*_pulse_ = 0.02 V, *E*_condition_ = −0.4 V, *E*_deposition_ = −1 V, frequency = 50 Hz, deposition time = 300 s and equilibrium time = 20 s.

SPAN has a good electrical conductivity in acid solution, and acetate buffer solution (10 mM, pH 4.6) is usually used for Pb(II) and Cd(II) determination by ASV using bismuth film electrodes [1,2,13]. Therefore, acetate buffer solution (10 mM, pH 4.6) was employed as the supporting electrolyte. The effect of Bi^3+^ concentration was investigated, due to the thickness of bismuth film formed by Bi^3+^ influencing the formation of binary or multicomponent “fusible” alloys with various heavy metals, which in turn affects the currents response [2]. As seen in Figure 3A, the stripping peak current of Cd^2+^ and Pb^2+^ increased with the Bi^3+^ concentration ranging from 100 μg·L^−1^ to 500 μg·L^−1^. Besides, no obvious increase of the peak current was found over 300 μg·L^−1^ of the Bi^3+^ concentration, which was considered as the saturation of bismuth film. Meanwhile, the higher Bi^3+^ concentration would block the conductive surface of the electrode, reduce the number of active sites and cause competitive enrichment between Bi^3+^ and target metals on the electrode surface [2,31]. However, in the case of Bi^3+^ 100 μg·L^−1^, probably the thickness of the film is not sufficient. Thus, for further studies, the Bi^3+^ concentration of 300 μg·L^−1^ was selected.

Besides, it was found that a suitable salt concentration can enrich Bi^3+^ and target metals on the electrode surface and, thus, increases the current response. When the sensor was tested with 10 mM acetate buffer solution (pH 4.6) containing 300 mM NaCl, the current response reached plateau and then remained stable (see Figure 3B). Therefore, in this case, we chose 300 mM NaCl as a compromise among the high sensitivity, good resolution and reproducibility of the peaks.

#### 2.3.2. Effect of Deposition Time

The pre-concentration time usually affects the sensitivity of the sensor; in fact, lower detection limits can be obtained with longer deposition time. In our case, we investigated a deposition time range between 300 s and 900 s, with Cd^2+^ and Pb^2+^ both at a concentration of 10 μg·L^−1^. As expected (Figure S1), by increasing the deposition time, there is an increase of sensitivity for each heavy metal tested. However, for the further experiments, 600 s was chosen for a compromise between the sensitivity and speed of the measurement. The relatively long deposition time may be ascribed to the palm-sized electrochemical workstation due to its potential in portable applications, and a shorter time can be applied when using regular-sized instrument with higher precision.

#### 2.3.3. Effect of Frequency, SW Pulse Height and SW Step Increment

**Figure 4:**
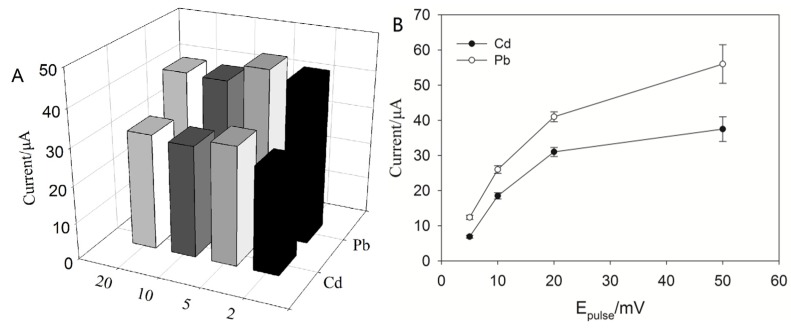
(**A**) Frequency effect on peak height in a solution containing 300 μg·L^−1^ Bi^3+^, 10 μg·L^−1^ Pb^2+^ and Cd^2+^ and 300 mM NaCl. SWASV parameters: *E*_begin_ = −1 V, *E*_end_ = −0.4 V, *E*_step_ = 0.010 V, *E*_pulse_ = 0.02 V, *E*_condition_ = −0.4 V, *E*_deposition_ = −1 V, frequency = 1–100 Hz, deposition time = 600 s and equilibrium time = 20 s. (**B**) Optimization of pulse potential (*E*_pulse_) using a solution containing 300 μg·L^−1^ Bi^3+^, 10 μg·L^−1^ Pb^2+^ and Cd^2+^ and 300 mM NaCl. SWV parameters: *E*_begin_ = −1 V, *E*_end_ = −0.4 V, *E*_step_ = 0.010 V, *E*_pulse_ = 0.005–0.05 V, *E*_condition_ = −0.4 V, *E*_deposition_ = −1 V, frequency = 100 Hz, deposition time = 600 s and equilibrium time = 20 s.

The effect of frequency was investigated by studying the peak height while varying the frequency in a range between 10 and 100 Hz. We observed an increase of peak height for both heavy metals at increasing frequency (see Figure 4A). The reason may be that the oxidation of these metals became less reversible at higher frequencies, which also also found by Kefala *et al.* [32] and Arduini *et al.* [11]; thus, in our case, a frequency of 100 Hz was selected for the rest of the work.

Pulse potential (*E*_pulse_) was also optimized by varying the SW in a range between 5 and 50 mV to investigate the SW pulse height. As seen in Figure 4B, a relevant increase in the interval from 0.005 to 0.05 V was observed. However, a pulse potential of 20 mV was eventually chosen for the compromise among high sensitivity, good resolution and reproducibility of the peaks (see Figure 4B). The step potential effect on the cadmium and lead ion peak height using a step increment from 0.001 to 0.02 V was studied, and an increase of sensitivity was observed for both heavy metals tested (Figure S2). However, in this case, we also chose 0.01 V as a compromise among the high sensitivity, good resolution and reproducibility of the peaks current (Figure S2).

#### 2.3.4. Effect of Equilibrium Time

The equilibrium time is the time between the deposition step and the dissolution of the heavy metals reduced on the surface of the electrode in the deposition step by means of square wave voltammetry. The equilibrium time was investigated in order to obtain well-resolved peaks, where the heights of the cadmium and lead peaks remained almost unchanged (Figure S3). However, using 20 s as the equilibrium time, well-resolved and reproducible peaks were obtained (RSD% equal to 3.2% and 4.4% for Cd^2+^ and Pb^2+^, respectively), and thus, 20 s were chosen as the equilibrium time in the rest of the work.

### 2.4. SWASV Analysis of Cd^2+^ and Pb^2+^

**Figure 5:**
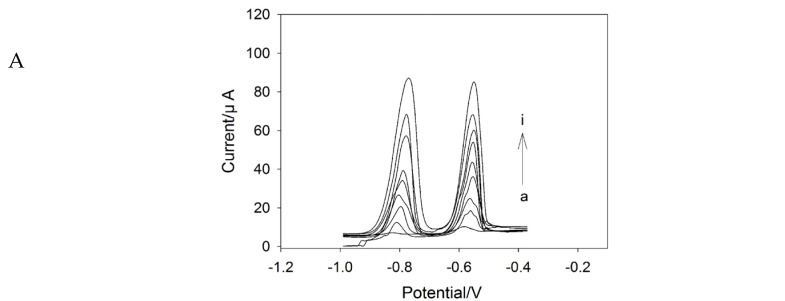

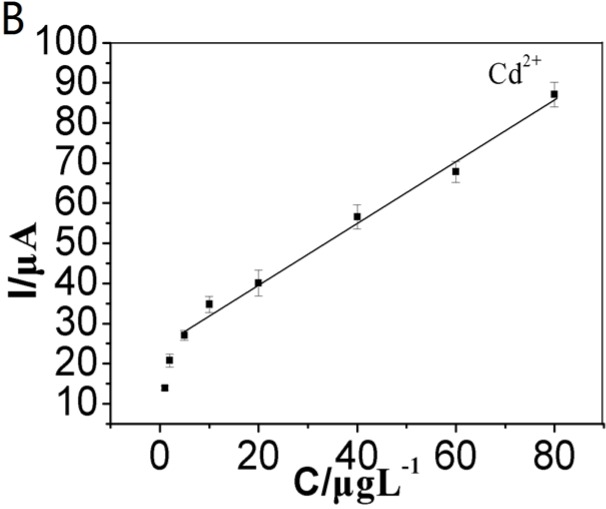

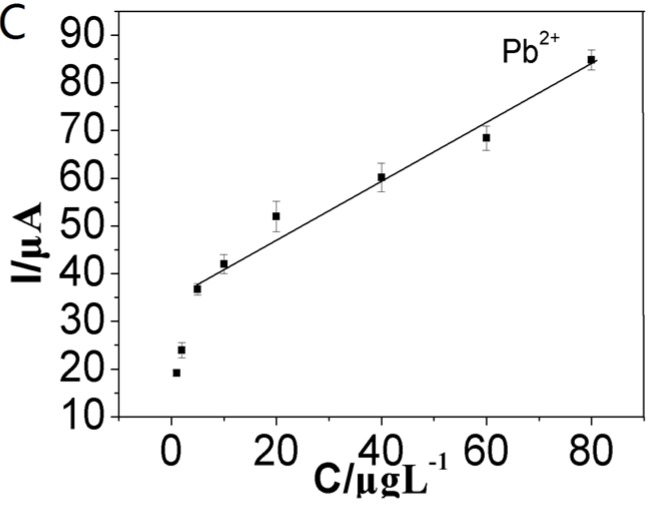
(**A**) SWASV curves at GCE/SPAN/MCN in 10 mM acetate buffer (pH 4.6) containing 300 mM NaCl, 300 μg·L^−1^ Bi^3+^, with different Cd^2+^ and Pb^2+^ concentrations (from a–i: 0, 1, 2, 5, 10, 20 , 40, 60, 80 μg·L^−1^). The SWASV parameters were *E*_begin_ = −1 V, *E*_end_ = −0.4 V, *E*_step_ = 0.010 V, *E*_pulse_ = 0.02 V, *E*_condition_ = −0.4 V, *E*_deposition_ = −1 V, frequency = 100 Hz, deposition time = 300 s and equilibrium time = 20 s. (**B**) and (**C**) show the plots of stripping peak current *vs.* the Cd(II) and Pb(II) concentration.

The SWASV responses of GCE/SPAN/MCN to Cd(II) and Pb(II) were investigated by increasing the metal ion concentrations from 1 μg·L^−1^ to 80 μg·L^−1^ under the optimum conditions described above. The analysis procedure was described in Section 2.4. As seen in Figure 5, the peak currents increased, and the peak potentials shifted little with the increase of the concentrations of Cd(II) and Pb(II). Figure 4 (inset) shows that the resulting calibration plots were linear over the range from 5 μg·L^−1^ to 80 μg·L^−1^. The calibration equation for Cd(II) was *I*p = (0.76948 ± 0.03551)·*C*_Cd_ + (24.2304 ± 1.14382) with a high correlation coefficient of 0.98944, and the calibration equation for Pb(II) was *I*p = (0.61772 ± 0.03316)·*C*_Pb_ + (34.5145 ± 1.27773) with a high correlation coefficient of 0.98576. The detection limits for Cd(II) and Pb(II) were calculated to be 0.7 μg·L^−1^ and 0.2 μg·L^−1^, respectively, based on signal-to-noise ratio of three (S/N = 3). The Cd(II) and Pb(II) detection performances of the proposed sensor were compared with other previously-reported Bi film-modified electrodes, and the results are listed in Table 1. Compared to the sensors based on Bi/SWNTs, Bi/Au-GN-Cys, Bi/2,2-azinobis (3-ethylbenzothiazoline-6-sulfonate) diammonium salt (ABTS)-MWCNTs, polymer/Bi and Nafion/Bi, this sensor exhibited an improved linear detection range. Besides, compared to the sensors based on reduced graphene oxide (RGO)/Bi and polymer/Bi, this sensor showed a relatively lower detection limit. All of these results provide evidence indicating that this sensor exhibited improved analytical performance in terms of linear detection range and showed a relatively lower detection limit. Additionally, the reason may be ascribed to the use of MCN and SPAN, which can provide fast electron transfer, a large surface area and more binding sites with heavy metal ions.

**Table 1:** Comparison of the analytical performance of some Bi film-modified electrodes for measurements of Cd(II) and Pb(II).

Electrode	Analytical Technique	Linear Range (μg·L^−1^)		Detection Limit (μg·L^−1^)		Reference
		Cd(II)	Pb(II)	Cd(II)	Pb(II)	
Bi/SWNTs/GCE	SWASV	0.5–11	0.5–11	0.076	0.18	[33]
Bi/Au-GN-Cys/GCE	SWASV	0.50–40	0.50–40	0.10	0.05	[1]
Bi/ABTS-MWCNTs/GCE	DPSV	0.5–35	0.2–50	0.2	0.1	[5]
Bi/Nafion/PANI-MES/GCE	SWASV	0.1–20	0.1–30	0.04	0.05	[34]
RGO/Bi/GCE	SWASV	20–120	20–120	2.8	0.55	[35]
polymer/Bi/GCE	SWASV	2–60	2–60	2	2	[36]
Nafion/Bi/GCE	SWASV	1–20	1–20	0.1	0.1	[37]
SPAN/MCN/GCE	SWASV	5–80	5–80	0.7	0.2	This work

SWNTs: single-walled carbon nanotubes; Au-GN-Cys: gold nanoparticle-graphene-cysteine composite; ABTS: 2,2-azinobis (3-ethylbenzothiazoline-6-sulfonate) diammonium salt; MWCNTs: multi-walled carbon nanotubes; PANI-MES: polyaniline-2-mercaptoethanesulfonate; RGO: reduced graphene oxide.

### 2.5. Reproducibility, Stability and Interference

The reproducibility was investigated by preparing five proposed electrodes based on the above-mentioned procedure and applied to the determination of 10 μg·L^−1^ Cd^2+^ and Pb^2+^. Under optimum conditions, the RSDs of five independent electrodes were 4.74% for Cd^2+^ and 3.72% for Pb^2+^, indicating that the fabrication procedure was reliable and the proposed electrode had good reproducibility (Figure S4). The electrode also had good long-term storage stability after one week of storage. All of the results showed the good performance of the proposed sensor.

The anti-interference ability of the modified electrode is crucial for the electrode’s practical application. In order to examine the anti-interference ability of the proposed sensor, various cations and anions were added to the acetate buffer solution containing 10 μg·L^−1^ Cd^2+^ and Pb^2+^ for SWASV analysis under optimum conditions. The results showed that a 100-fold concentration ratio of Al^3+^, Ca^2+^, Mg^2+^, Cl^−^ and K^+^, a 50-fold concentration ratio of Co^2+^, CO_3_^2−^ and Zn^2+^ and a 20-fold concentration ratio of Fe^3+^, Hg^2+^ and Ni^2+^ presented less than a 5.6% peak current decrease. However, Cu^2+^ has a relatively large influence. A 20-fold concentration ratio of Cu^2+^ presented a 13.2% peak current decrease; while a 10-fold concentration ratio of Cu^2+^ resulted in less than a 6.1% peak current decrease. Therefore, the result that the common ions had little effect on the quantification of Cd^2+^ and Pb^2+^ indicated a good anti-interference ability of the proposed sensor.

### 2.6. Application to Real Samples

The proposed sensor was used to quantify Cd^2+^ and Pb^2+^ in real samples with the purpose of evaluation the application of it. The real samples were collected from Taozi Lake, Changsha, China, and were filtered with a 0.45-mm membrane (purchased from Millipore, Boston, MA, USA), adjusted to pH 4.6 using acetate buffer with 300 μg·L^−1^ Bi^3+^ added. The recovery was found to be in the range from 96.48% to 104.15%, and the measurement results obtained by the proposed sensor were also in good agreement with the ICP-MS method (Table 2). The comparative result showed that the proposed sensor has good accuracy and recovery, suggesting the great application potential in real samples.

**Table 2:** Determination of Cd^2+^ and Pb^2+^ in real samples.

Sample	Added (μg L^−1^)		ICP-MS (μg L^−1^)		Proposed Sensor (nM)		Relative Concentration Deviation (%)	
	Cd^2+^	Pb^2+^	Cd^2+^	Pb^2+^	Cd^2+^	Pb^2+^	Cd^2+^	Pb^2+^
River Water 1	5	5	5.3 ± 0.26	6.1 ± 0.43	5.5 ± 0.66	6.5 ± 0.53	2.60	4.49
River Water 2	10	10	11.1 ± 0.96	10.9 ± 1.2	10.5 ± 0.49	11.3 ± 1.3	3.93	2.54
River Water 3	15	15	16.4 ± 1.9	17.5 ± 2.1	15.1 ± 1.4	16.8 ± 1.7	5.37	2.89

## 3. Experimental Section

### 3.1. Reagents, Preparation of SPAN Nanofibers and MCN

MCN was synthesized using SBA-15 (an ordered hexagonal mesoporous silica template) as a template, and carbon tetrachloride was used as a carbon source. SBA-15 and MCN were synthesized in our lab according to the method reported by Vinu and co-workers [38], with slight alterations [20,39]. Detailed information of the preparation of SPAN nanofibers is described in the Supporting Information.

Cd(NO_3_)_2_, Pb(NO_3_)_2_ and Bi(NO_3_)_3_·5H_2_O were used to prepare the stock solutions of Cd^2+^, Pb^2+^ and Bi^3+^ with ultrapure water, respectively, and the solutions were stored at 4 °C. Cetyltrimethylammonium bromide and aniline (AN) were purchased from Sinopharm Chemical Reagent Co., Ltd. (Shanghai, China). Acetate buffer solution (10 mM, pH 4.6) was prepared by CH_3_COOH and CH_3_COONa and used as the supporting electrolyte. All chemicals were of analytical grade and used as received; ultrapure water was used for the preparation of all the solutions.

### 3.2. Apparatus

Cyclic voltammetry (CV) measurements and square wave anodic stripping voltammetry (SWASV) were carried out using a portable PalmSens Instrument (CHI1230B A14535 PalmSens, Utrecht, The Netherlands) connected to a personal computer. In this work, the electrode system consists of three parts. GCE/SPAN/MCN was proposed as the working electrode, saturated calomel electrode (SCE) and Pt foil were used as reference electrode and auxiliary electrode, respectively. Scanning electron microscopy (SEM) of the materials was obtained with an S-4800 scanning electron microscope (Hitachi Ltd., Tokyo, Japan). Transmission electron microscopy (TEM) was obtained with a Tecnai G2 F20 S-TWIX electron microscope (FEI, Eindhoven, The Netherlands). A Model PHSJ-3F laboratory pH meter (Leici Instrument, Shanghai, China) was employed for pH measurements of the buffer solutions.

### 3.3. Preparation of the Modified Electrodes

The bare glassy carbon electrode (GCE) was polished in alumina slurry and then rinsed with deionized water. After being rinsed with ultrapure water, the electrode was sonicated in acetone, ethanol and water successively [40,41]. Then, the electrode was electrochemically treated in 0.5 M H_2_SO_4_ by cyclic voltammetry between −0.5 V and 1.5 V at 50 mV·s^−1^ until a steady-state redox wave was observed. The self-assembled film was modified onto the GCE surface by layer-by-layer means. First, 5 μL of the 2 mg·L^−1^ SPAN nanofiber suspension (dispersed in DMF) was dripped onto the clean electrode surface, marked as the GCE/SPAN. After electrode drying, 5 μL of MCN suspension (dispersed in DMF) were dip-coated on the GCE/SPAN, and dried in air to obtain GCE/SPAN/MCN. Then, the prepared electrode was copiously rinsed with acetate buffer solution. When not in use, the electrode was stored in a moist state at 4 °C.

### 3.4. Heavy Metal Measurement Using the Electrochemical Sensor

The modified electrode (GCE/SPAN/MCN) was immersed into 10 mL acetate buffer solution of pH 4.6, containing 300 μg·L^−1^ of bismuth, and standard lead and cadmium solutions were added. The analytical measurements were carried out in SWASV mode. Voltammetric experiments consisted of three conventional steps: time-controlled electrochemical metal deposition, rest period and a positive-going voltammetric stripping scan under the chosen conditions. In some cases, it was followed by a forced electrochemical conditioning (cleaning) step with stirring solution to remove the target metals eventually staying on the working electrode. The SWASV parameters are: *E*_begin_ = −1 V, *E*_end_ = −0.4 V, *E*_step_ = 0.010 V, *E*_pulse_ = 0.02 V, *E*_condition_ = −0.4 V, *E*_deposition_ = −1 V, frequency = 100 Hz, deposition time = 600 s and equilibrium time = 20 s. All measurements were carried out in acetate buffer (10 mM, pH 4.6) with NaCl (300 mM) as the electrolyte.

## 4. Conclusions

An SPAN/MCN/GCE has been successfully developed and used for simultaneous voltammetric determination of trace Cd^2+^ and Pb^2+^ by the SWASV method. The excellent properties of MCN and SPAN contributed to the superior performance of the proposed electrode, such as high electrochemical sensitivity, lower detection limit, good capability of anti-interference and improved potential in real samples. Novel materials based on SPAN would be an interesting research topic, and the combination of carbon materials and SPAN in electrode fabrication would give us an alternative route to develop new types of electrodes for heavy metals determination.

## Supplementary Materials

The following are available online at http://www.mdpi.com/2079-4991/6/1/7/s1.
