# Supplementary material for: Determination of Cd2+ and Pb2+ Based on Mesoporous Carbon Nitride/Self-Doped Polyaniline Nanofibers and Square Wave Anodic Stripping Voltammetry
Source: Nanomaterials (Basel). 2016 Jan 4;6(1):7. doi: 10.3390/nano6010007 (PMC5302537; doi:10.3390/nano6010007)
Supplement: Supplementary file 1 [file nanomaterials-06-00007-s001.pdf]

Supplementary Materials

Preparation of SPAN Nanofibers

SPAN nanofibers were prepared according to the method provided by Zhang and co-workers [1]. Briefly, 0.11 g of cetyltrimethyl ammonium bromide (CTAB), 0.23 g of aniline (AN), 0.43 g of 2-aminobenzenesulfonic acid (ABS) were dissolved in 40 mL 0.15 M HCl solution. Then, 20 mL 0.06 M ammonium peroxydisulfate (APS) was added to the mixed solutions. The copolymerization reaction was performed under static conditions at 5 °C for 24 h. Finally, the resulting dark green precipitates were filtered, then, washed with ultrapure water and ethanol several times, and dried at room temperature.

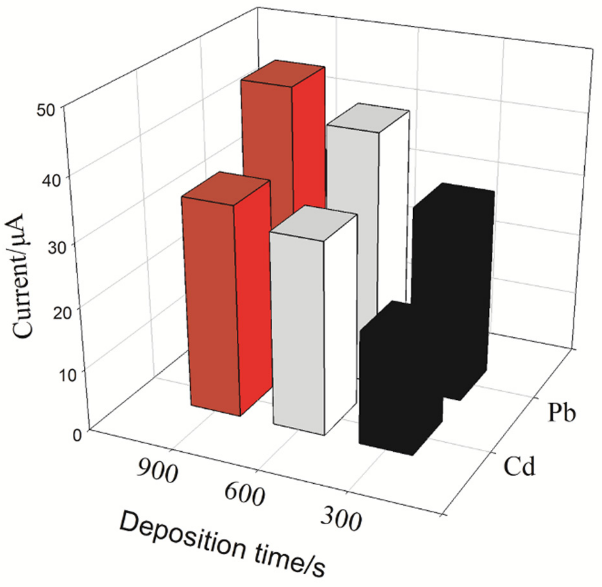

Figure S1. Deposition time effect on peak height in a solution containing 300 μg·L^{-1} Bi^{3+}, 10 μg·L^{-1} Pb^{2+} and Cd^{2+}, and 300 mM NaCl. Square wave anodic stripping voltammetry (SWASV) parameters: $E_{begin} = -1$ V, $E_{end} = -0.4$ V, $E_{step} = 0.010$ V, $E_{pulse} = 0.02$ V, $E_{condition} = -0.4$ V, $E_{deposition} = -1$ V, Frequency = 50 Hz, deposition time = 300–900 s, and equilibrium time = 20 s.

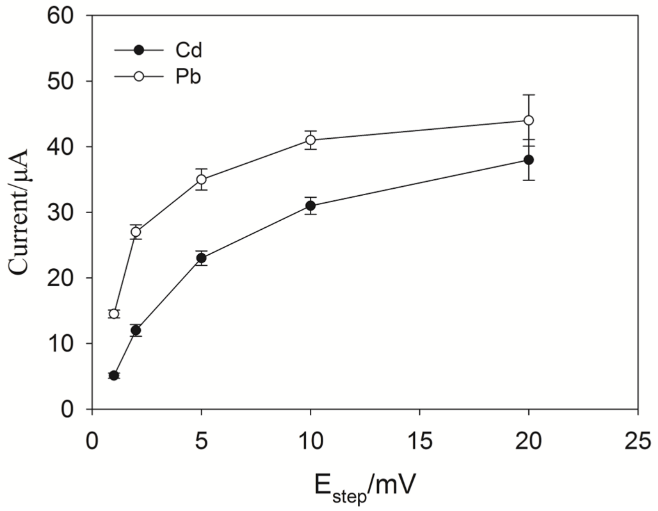

Figure S2. Optimization of step potential effect (E_{step}) using a solution containing 300 μg·L^{-1} Bi^{3+}, 10 μg·L^{-1} Pb^{2+} and Cd^{2+}, and 300 mM NaCl. SWASV parameters: $E_{begin} = -1$ V, $E_{end} = -0.4$ V, $E_{step} = 0.001$–0.02 V, $E_{pulse} = 0.02$ V, $E_{condition} = -0.4$ V, $E_{deposition} = -1$ V, Frequency = 100 Hz, deposition time = 600 s, and equilibrium time = 20 s.

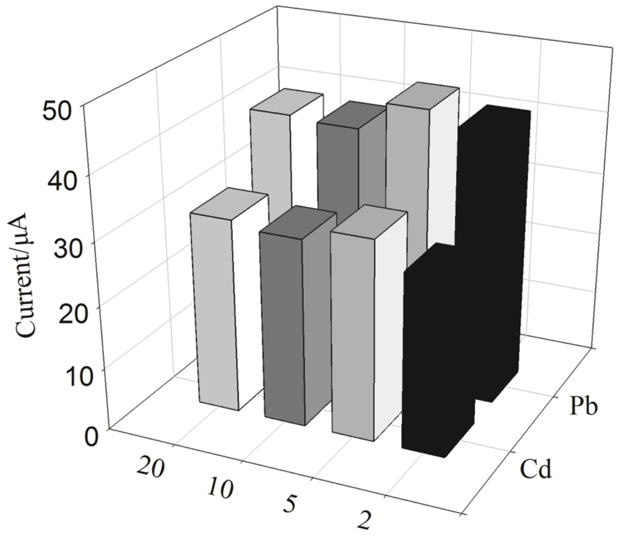

Figure S3. Equilibrium time effect on peak height in a solution containing 300 μg·L^{-1} Bi^{3+}, 10 μg·L^{-1} Pb^{2+} and Cd^{2+}, and 300 mM NaCl. SWASV parameters: E_{begin} = −1 V, E_{end} = −0.4 V, E_{step} = 0.01 V, E_{pulse} = 0.02 V, $E_{condition}$ = −0.4 V, $E_{deposition}$ = −1 V, Frequency = 100 Hz, deposition time = 600 s, and equilibrium time = 2–20 s.

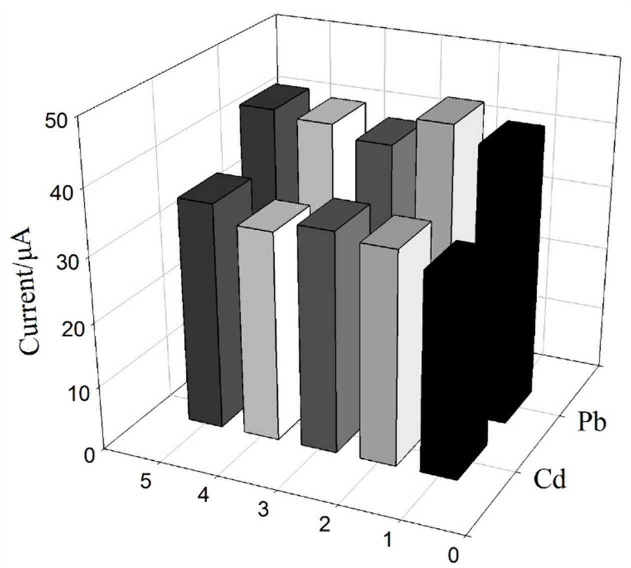

Figure S4. The reproducibility of proposed electrodes.

Reference

1. Zhang, C.; Li, G.; Peng, H. Large-scale synthesis of self-doped polyaniline nanofibers. *Mater. Lett.* **2009**, *63*, 592–594.

© 2016 by the authors; licensee MDPI, Basel, Switzerland. This article is an open access article distributed under the terms and conditions of the Creative Commons by Attribution (CC-BY) license (http://creativecommons.org/licenses/by/4.0/).
